# Perceptions and Attitudes of Informal Caregivers of Stroke Patients Regarding the *Stroke-CareApp*: A Phenomenological Study

**DOI:** 10.3390/healthcare13172082

**Published:** 2025-08-22

**Authors:** Ismael Andrades-González, Neiva Rodríguez-Estrabot, Rocío Magdaleno-Moya, Jesús Molina-Mula

**Affiliations:** 1 Son Espases University Hospital, Carretera de Valldemossa, 79, 07120 Palma, Spain; neiva.rodriguez@ssib.es (N.R.-E.); rocio.magdaleno@ssib.es (R.M.-M.); 2 Nursing and Physiotherapy Department, University of Balearics Island, 07122 Palma, Spain; jesus.molina@uib.es

**Keywords:** caregivers, stroke, e-health

## Abstract

**Introduction**: The application of information and communication tools in healthcare is becoming increasingly widespread and is obtaining promising results. However, their use by informal caregivers is not adequately elucidated. **Objective**: The aim was to analyze the opinions, perceptions, and attitudes of informal caregivers of stroke patients concerning the use of *Stroke-CareApp* (Version 1), a smartphone application (app) designed exclusively for this population. **Methods**: A qualitative study was conducted using a phenomenological approach. Five caregivers used *Stroke-CareApp*, an app designed as a meeting place for peers, with information about the disease and access to healthcare professionals. **Results**: The discourses obtained from the interviews were analyzed, and the resulting codes were divided into eight categories: impact on the caregiver, coping with caregiving, involvement in caregiving, steps toward recovery in the absence of the caregiver, relevance for the caregiver, facilitating factors for the use of the app, source of consultation when in doubt and reliability of the information, and limitations in the use of the app. **Conclusions**: Although caregivers consider the app a beneficial intervention for them, it is important to note that it is a complementary alternative to other interventions, and one must be patient and perseverant during the initial months to achieve optimal adherence.

## 1. Introduction

Information and communication technology (ICT) tools have become an integral part of people’s lives and even changed the way we communicate. In 2022, almost two-thirds (66.3%) of the world’s population were using the internet. Furthermore, in Spain, 96.1% of households had internet access, where 16.2% of these users had used telemedicine services, with the smartphone being the star device through which they accessed the services (87.7%) [1].

The field of telemedicine makes health services more accessible and approachable for the entire population while saving costs and positively impacting the health of individuals and communities. To that end, a variety of mobile applications (apps) have been designed for smartphones targeting different types of patients depending on the pathology they suffer from [2].

Despite the existence and rising prevalence of these apps, a recent systematic review showed that informal caregivers of stroke patients lacked this type of resource [3]. The number of stroke patients is on the rise in Europe and in Spain, with 40,000 reported cases each year. Of these, more than half (52.5%) exhibit a significant degree of dependence that requires the care of their relatives, who become informal or family caregivers [4].

Most existing stroke apps are geared toward clinical patient monitoring, focusing on aspects related to treatment, assessment, or healthy habits, without comprehensively addressing the needs of informal caregivers [5,6].

There are some apps that devote space to caregivers, such as ICTUSCAT in Catalonia, which offers a mobile app and web platform that combines personalized notifications, stroke sign recognition, health data monitoring, and a direct communication channel between caregivers, patients, and healthcare professionals [7]. Living at home in Andalusia allows caregivers to remotely manage household tasks, medication, and calls [8]. At the national level, there are platforms such as Jointalk and Carely that act as an emotional social network for sharing experiences [9]. The app analyzed in this study integrates these three key functionalities in an unprecedented way—a meeting place for peers, specialized information about the disease, and direct access to healthcare professionals—and therefore represents a digital innovation with high potential to improve the well-being of informal caregivers.

Informal caregivers pose challenges for the healthcare system for two reasons: on the one hand, they represent an important replacement in the healthcare costs of all countries [10], and, on the other hand, they generate new care demands on the healthcare system, since their rhythm of life is altered, causing emotional alterations, loss of social relationships, appearance of new diseases, and, in short, a deterioration of health and loss of quality of life [11,12].

In light of the above, it is important to be aware that informal caregivers form a critical user group of the healthcare system. Thus, making these services more accessible and approachable for them through telemedicine is a beneficial alternative, since studies have demonstrated a positive effect on caregivers when applying e-health-based interventions by healthcare personnel [13,14,15,16]. In addition, it is evident that the use of different ICT tools has the most notable impact at home, as it is the environment wherein family problems often surface owing to the lack of support felt by caregivers [17,18].

Despite the benefits and advantages of ICT tools and, specifically, telenursing with health system users, there is not much evidence describing their use by informal caregivers of stroke patients. Therefore, it would be advisable to analyze the opinions that these caregivers have about this tool and whether there are factors that limit or facilitate its use.

Thus, this study aimed to analyze the opinions, perceptions, and attitudes of informal caregivers of stroke patients concerning the use of a smartphone app designed exclusively for this population.

## 2. Materials and Methods

### 2.1. Design

A qualitative methodology based on the constructivist paradigm was used for this study. A phenomenological approach was adopted to analyze the opinions, perceptions, and attitudes of informal caregivers of stroke patients regarding the use of a mobile app designed for them.

In this sense, although opinions (value judgments), perceptions (subjective interpretations), and attitudes (behavioral predispositions) may be three distinct concepts, separating them in this type of methodology and design is not always possible, as they are elements closely linked to human experience [19]. Furthermore, the main objective lies in understanding subjective experience in its complexity and from the perspective of the participants [20], and the researcher seeks to find “situated meanings” rather than measuring isolated constructs [19].

### 2.2. Theoretical Perspective

The phenomenological approach is considered ideal to achieve the objective set out in this study, since, according to Husserl, it aims to explain the essence and veracity of phenomena, as well as the nature of things, in order to reach an understanding of the lived experience in its complexity [21].

The analysis and presentation of the results of this study are based on a phenomenological–hermeneutic or interpretative reflection proposed by Heidegger. From this perspective, an attempt is made to understand the experience lived by a person while accounting for the existing similarities and differences in the meanings of these experiences [22,23,24].

In addition, elements of Husserl’s descriptive phenomenology will also be used, since, when combined with Heidegger’s phenomenology, the aim is to achieve complementarity between the two, which in a phenomenology based on empirical research can begin with a rigorous description of reality and then move on to a situated interpretation [25,26,27]. This combination allows for a more in-depth and rich analysis of the data obtained, maintaining phenomenological fidelity and broadening the hermeneutic understanding of the phenomenon.

Finally, to interpret the results, elements from the works of Bandura [28], Bourdieu [29], and Orem [30] were incorporated to rigorously present the discussion of the results.

### 2.3. Participants

The study was performed at the tertiary-level Hospital Universitario Son Espases, located in Palma de Mallorca, Spain. This center serves a population of 1,127,998 inhabitants and contains the stroke unit, which treats about 800 new cases of stroke annually in the Balearic Islands [31].

For this study, five informal caregivers (ICs) of stroke patients were interviewed following the principles of purposive sampling, giving priority to the depth of description and interpretation of the data [32]. Once data saturation was reached, understood as the point at which the interviews did not provide new categories or relevant topics [33], the interviews were discontinued when the codes and meanings in the last two interviews substantially coincided with the previous ones. The following selection criteria were followed:

- Agreed to participate in the study and signed the informed consent form.
- Informal caregivers of stroke patients with a Barthel index < 60.
- Have a smartphone and know how to use it.
- Over 18 years of age.

The caregivers were contacted by a key informant, and subsequently, the principal investigator (PI) contacted the caregivers in person at the inpatient unit. Once face-to-face contact was established between the PI and the ICs interested in participating in the study, the study was explained to them, and the app was presented to them. In addition, a date was set after six months to conduct the interviews.

At the request of the ICs themselves and to facilitate reconciliation with caregiving, the interviews were conducted through video calls. The first interview was conducted on 15 February 2024 and the last on 8 March 2024. Of the six participants interested in participating at the beginning of the study, one withdrew from the study when contacted for the interviews for personal reasons.

### 2.4. Data Collection

This study employed two data collection techniques: interviews and the field diary maintained by the PI.

On the one hand, a conversational technique was used through semi-structured in-depth interviews lasting between 20 and 30 min. The analysis of the participants’ discourse allows for the interpretation of the different meanings they may have according to their social and cultural customs and influences [34].

In relation to the semi-structured interview as a data collection tool, it allows us to guide the conversation with participants while leaving room for spontaneous responses and unexpected nuances to emerge.

Thus, as Hernandez Sampieri [20] points out, this type of tool is useful for the study’s objective of understanding experiences and meanings, as participants can integrate opinions, perceptions, and attitudes into their discourse in a natural way. Furthermore, it should be considered that attitudes include beliefs and perceptions [35], and as Flick [19] points out, in qualitative work, analytical categories are not always rigidly defined before data collection but may emerge in interaction with the participant.

During the initial contact, a series of sociodemographic data were collected as well as the dependency status of the family member affected by the stroke using the Barthel scale and informed consent. At the beginning of the interviews, participants were asked whether any of the sociodemographic data collected previously had changed, modifying only the patient’s Barthel index. In addition, the previously signed informed consent was recalled, obtaining confirmation. The sociodemographic data of the participants are presented in Table 1.

On the other hand, the PI used a field diary during the course of the study, which consisted of notes, written or audio, with observations that the PI perceived about the ideas, emotions, or thoughts of the participants or of the PI themselves. This was intended to improve the external and internal validity of the study [36].

### 2.5. Stroke-CareApp

*Stroke-CareApp* is a smartphone application designed to implement this project. The design and content of the app were implemented by the research group and a panel of collaborators who were experts in the field using the Delphi method [37]. The development of the app was carried out by a group of computer scientists coordinated by the PI of the project. To see the design and user interface, please visit the following link: http://www.play4health.com/app/ictus/help/index.html.

The app consisted of three different parts. The first part featured Frequently Asked Questions (FAQs) with general information about the disease and the new situation that caregivers will have to face. The second part had a forum that promoted the relationship between caregivers, and thus, they could share information and advice among themselves. The third part allowed them to contact the research team (nurses, physiotherapists, neurologists, and social agents) from the application itself.

The first block of content for caregivers was structured into sections that addressed the impact of stroke, its risk factors, clinical manifestations, and sequelae. Each section was complemented with infographics and explanatory animated videos to facilitate understanding. Specific material for caregivers included general advice and videos produced by the research team on patient mobilization techniques, aimed at preventing injuries to both the patient and the caregiver. Information was also provided on the assistance available at the national and regional levels, with direct links to these services, as well as a list of associations that could be contacted for support and a compendium of complementary bibliographic material.

The forum, which was exclusively accessible to caregivers, allowed them to interact with each other through open questions or the publication of personal experiences that could be helpful to other participants. From the outset, clear rules were established regarding the type of content allowed, appropriate language, and expected behavior. The forum was moderated by the research team, which was responsible for reviewing and supervising messages, intervening when necessary, and promoting a constructive environment, maintaining the organization and purpose of the space.

Ultimately, the objective of the app was to promote the empowerment of the caregivers through the most interesting information for them and the approach to health professionals without having to travel, thus avoiding the consequences at an economic, labor, or care-provision level that such displacement may cause.

### 2.6. Data Analysis

The data analysis was based on an iterative, inductive, and complex process comprising several phases that resulted in the PI becoming involved in the narratives and descriptions put forward by the participants in order to understand their experience [34,36].

This process was carried out using open coding, which allowed us to capture the diversity of experiences and perceptions of the participants through an in-depth reading in which relevant units of meaning were identified without preconceived categories. Codes and categories were then developed collaboratively by discussing and agreeing on the meanings attributed to the texts and developing a code book that was progressively refined. Once this phase was completed, a thematic refinement was carried out from which the main themes were derived through a process of conceptual grouping and constant review. In addition, discrepancies were resolved jointly by applying the criteria of internal consistency and relevance to the objectives. Finally, cross-checks were carried out between coders, prioritizing reflective discussion and consensus as a validation strategy.

Due to the small number of interviews, no software tools were used; therefore, the information from the interviews was captured by an audio recorder and then transcribed using the online tool “Sonix”. Each transcript was reviewed by the PI.

The transcripts obtained were anonymized by assigning an acronym and a number to each interview. Then, the transcribed texts were read, underlining and commenting on the most interesting parts. After several readings, the information obtained was systematically coded.

Once this work was completed, the information extracted from the texts was coded, marking words, sentences, or complete paragraphs to identify specific themes and identify the significant aspects.

Subsequently, a descriptive phase of the codes was carried out, and they were grouped into categories and subcategories, where the descriptions and interpretations of all the information processed and analyzed were integrated and defined.

**Figure 1:**
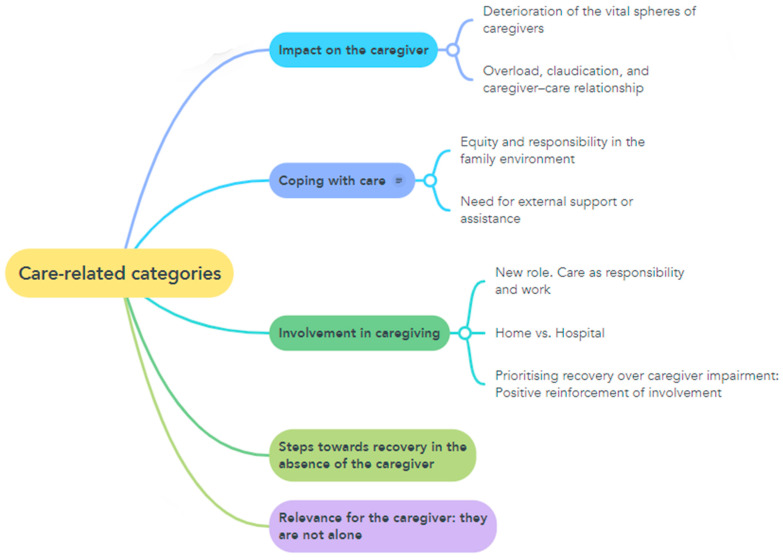
Care-related categories.

**Figure 2:**
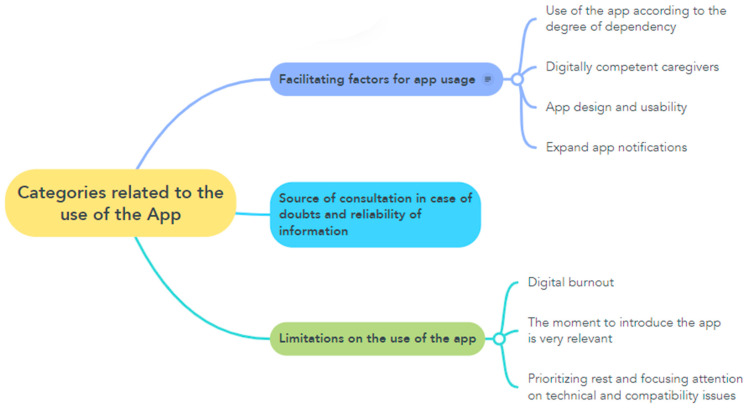
Categories related to the use of the app.

Figure 1 and Figure 2 reflect the categories, subcategories, and codes obtained in the course of the analysis of the study data.

Because the PI played multiple roles in the study (application development, direct contact with participants, data collection, and primary analysis), and recognizing that this could influence the interpretation of data and interaction with participants, in order to mitigate potential biases, a reflective journal was maintained throughout the process in which observations and methodological decisions, preconceptions, personal reactions, and reflections on positioning were recorded.

Finally, to support the reliability and credibility of the study, three of the five interviews and the concept maps obtained by the PI were triangulated with two other members of the research team (JMM and NRE). These three interviews were selected based on the criteria of thematic saturation and contextual relevance, as they offered the most representative elements in relation to the main objective. The remaining two interviews, although valuable, did not contribute any significant discrepancies or new categories. The purpose of this triangulation was to ensure that both the data collection process and the analysis were adequate and that therefore the results and conclusions obtained accurately reflected the reality studied [36].

### 2.7. Ethical Considerations

The participants of this study were informed both verbally and in writing through the information sheet, reading, and signing the informed consent. Any doubts they had regarding the study were resolved.

The information related to the participants was anonymized and retained by the PI, thus ensuring compliance with current legislation both in terms of data protection, as provided for by the Organic Law 3/2018 from December 5 on the Protection of Personal Data and Guarantee of Digital Rights, and Law 41/2002 on Patient Autonomy and Clinical Information and Documentation. In addition, the app and the data derived from it respected the General Data Protection Regulation (GDPR) from 25 May 2018.

This research was approved by the Research Ethics Committee of the Balearic Islands (CEI-IB) on 5 March 2021, with the favorable report number IB4364/20PI. It was also approved by the Ethics Committees of the Hospital Universitario Son Espases and the Primary Care Management of Mallorca.

The approvals of the various ethics committees were valid throughout the course of the research. Nevertheless, the various delays and modifications to the protocol were duly communicated and confirmed by the other party.

## 3. Results

The results obtained after the analysis of the participants’ discourses were grouped into different categories and subcategories. Each category and subcategory is explained and substantiated by the textual speeches of the participants. The fact that the categories of opinions, perceptions, and attitudes are not explicitly differentiated is because, as Flick [19] and Hernandez Sampieri [20] state, the complexity and richness of human experience are reflected in the interaction between these three categories, which cannot always be separated and delimited, and it is common in this type of research for them to be naturally intertwined in the participants’ discourse.

Furthermore, in order to avoid losing the holistic understanding of the phenomena analyzed, great care was taken not to artificially segment the responses into these categories, allowing them to emerge from the data [38].

The speeches are coded to preserve anonymity and will be identified with a C and a number that will belong to each caregiver.

### 3.1. Coping with Caregiving

The category of coping with caregiving encompasses how the new caregivers and the family cope with the new situation of dependency of their affected family member. This category is divided into two subcategories.

#### 3.1.1. Equity and Responsibility in the Family Environment

Within the family of the person who has suffered a stroke, it is important that the family structure is consistent, i.e., that there is a relationship of respect and trust among family members that allows for an optimal support system that offers care and stability among family members. This has an impact on the primary caregiver, as it allows for an equitable distribution of care and the best possible way of coping with the new situation brought about by the stroke. When there are several siblings who live close to each other and share an amicable relationship, care is easier to cope with, since each family member does not feel that the responsibility is solely his or her own and feels more liberated.

*Well, it’s good for now because the three brothers are taking turns and when one goes, another one comes, and we do it. While one has no work, he comes and substitutes for the other. Now there is one who is on vacation, while the other has started working so we alternate. One sleeps there some days, the other one sleeps there other days, and depending on how things go and the time needed, we do it…*

C2

*Look, we are three siblings, and there is my older brother, who is the one who has taken care of my dad the most because I left, I went to Mexico because my sister is working there. So I was there the first month that everything happened, that we were admitted, and the second month and the third month my brother took care of it because I was away. So, I came back in mid-February and now it’s my turn…*

C3

#### 3.1.2. Need for External Support or Assistance

In cases where there is no good family support, or even where there is, but the family members’ own pace of life, including work and family commitments, among others, makes it difficult to provide the care required by the family member, it becomes necessary to generate the capacity to seek external help to cover the needs that the caregivers cannot cover. This external help relieves the feeling of responsibility and guilt that grows in caregivers in these situations.

*My partner and I are alone, but we are lucky enough to work from home, so we can be at home and have some freedom in our schedules. When there is an event, we have to ask someone to stay with her so that we can do it…*

C1

*It breaks our hearts to leave her alone because she can’t even press the button to call the nurse (…). So, of course, since life goes on and we all have to continue working, in the end what we do is to hire a non-professional caregiver…*

C5

### 3.2. Impact on the Caregiver

This category addresses the effects of the caregiver’s new role on his or her daily life. This category is divided into two subcategories.

#### 3.2.1. Deterioration of the Vital Spheres of Caregivers

Caring for a person with stroke by a family member has a negative impact on most aspects of life of the caregiver.

There is a deterioration in social relationships, since they have to stop engaging in leisure activities and seeing their friends. Likewise, work and economic aspects are also affected, since caregivers have to dedicate part of the domestic economy to modify the house or buy utensils for their family member and cut back on work schedules.

In many cases, the relationship with their children and/or partner worsens because the time they spend caring for them is taken away as a result of being at home with their family member depending on their care.

*Radically in all spheres of my life. At the family level, because I hardly see my children. I have already told my friends not to be angry with me, that I will come back someday because I don’t have so much time. On an economic level, we had to fix the apartment because, of course, my mother was a very hard-working and independent person. And of course, all of a sudden, we realized that she couldn’t go back home just like that. So we had to buy crane beds…*

C5

*And the work that I have has been cut back because I used to work from 9 am to 5 pm, and now I work from 8 am to 2:30 pm. I have a relationship with a woman who lives in Bilbao, and she has also been affected. Well, in general, my diet has worsened, I don’t know anymore…*

C4

#### 3.2.2. Overload, Claudication, and Caregiver–Care Relationship

The feeling of not having time for oneself, being permanently tired, and the sadness of seeing one’s dependent relative are aspects that are repeated in the discourse of caregivers.

Notwithstanding, they also experience a sense of resignation, responsibility, and strength that keeps them going. Likewise, there is no worsening of the relationship with the dependent relative, and a discourse emerges that the care and the new situation, in some cases, even improve the relationship with the relative.

*I have almost no time for myself or to do my own things anymore. That is to say, having to be very limited in time, because of course, you have to change it three times, you have to give the food…*

C1

*The truth is that for my brother it has been very good because it has increased his confidence in my father. With my brother, their communication and empathy with each other have improved a lot. That has improved a lot. It’s the same with me because me and my dad have always lived together, and we already knew each other…*

C3

*On an emotional level, it does make me sad to see her like this, because she wouldn’t want to be like this, always talking to her. But look, these are things that you don’t decide, and you have to go on…*

C2

*I find myself permanently very tired…*

C4

### 3.3. Involvement in Caregiving

This category is divided into three subcategories and covers patients’ feelings about the new role and the differences that appear depending on where the patient is in the initial months after the stroke.

#### 3.3.1. New Role: Care as Responsibility and Work

Caregivers perceive the new situation as one more job, an almost exclusive dedication to their family member, and despite having help, they feel the responsibility to be present as much as possible in the care.

In addition, with the new role acquired come feelings of commitment to the family member, guilt over not being able to care for him/her as he/she would like, or, in other cases, an obligation.

*If you go out, you are always on the lookout, you have to come back, even if Pedro is there. But there are things that I have to do…*

C1

*And well, I work in the morning and come here in the afternoon, every month, seven days a week, coming here and taking care of my mother is a job in itself…*

C4

*But during the day, we are there all the time, we do all the hygiene, we change her diaper with my sister and my brother or the caregiver. Well, we do the postural changes, we are with her, we feed her, we give her dinner, everything…*

C5

#### 3.3.2. Home Versus Hospital

There is a difference between caregivers who go directly to the home or to the hospital.

In the case of going home, the family member is usually less dependent, and recovery times are faster. Therefore, the caregiver feels greater satisfaction, and the level of involvement is lower, since they understand that their role is not absolutely indispensable for the good recovery of the family member.

On the other hand, caregivers with hospitalized family members, who are usually more affected, show greater involvement. This is because they strive to learn everything they can, so that when they go home, where they feel more alone, they have better skills and knowledge that they lack.

*Because it seems like I have also become very involved in the issue of changing it, of learning… because I think I have to learn how to do these things. I don’t know, maybe I don’t need it, but if I need it, I’d better learn it already, right?…*

C4

*So, all I do is look for information to help my mother. Because of course, I’m not a doctor, and I don’t know much about it either. So I look for speech therapist videos to see what I can do to help her recover her voice. Also, with a view to the fact that I imagine that in two or three weeks we will be discharged, and then when we get home, I will be able to find out things…*

C5

#### 3.3.3. Prioritizing Recovery over Caregiver Impairment: Positive Reinforcement of Involvement

Involvement stands out among caregivers who have less family support and/or are admitted to the hospital. Seeing that the effort they make in these first months has a positive impact on their relatives comforts them and makes them participate in their relative’s recovery, even if this has a negative impact on their own health.

*So, I take it as my time is invested in my mother’s recovery. And well, if it costs me a physical or mental effort, well, I assume it…*

C4

*From time to time I don’t have the obligation to go, in quotation marks, right? From 3.30 pm to 10.00 pm at night, but I always go for a few hours…*

C5

### 3.4. Steps Toward Recovery in the Absence of the Caregiver

The evolution of the patient in these first months is one of significant dependence, and most of them are still in the hospital recovering. Patients who have recovered earlier are at home, and this means that the caregivers have been able to establish routines and feel more liberated. However, the insecurity over abandoning continuous care or the absence of a caregiver remains.

*Her level of dependency is so high… The orderlies come in the morning, and after we do her hygiene, the orderlies come and sit her in the chair she has, and we put her on the bed, but she can’t do anything. She has lost all mobility with the hemiplegia on the left side…*

C5

*She gets up from the chair and goes to bed. But well, this week she has already taken a big leap because she was starting to walk between bars and well, the truth is that she is in better spirits. Her speech is affected, and her memory is still a little bit there… quite resentful…*

C4

*He now showers by himself, tidies his bathroom by himself… He is in a wheelchair, but they have already given him a cane so that, with company, he can walk little by little, and he is in this process. He eats alone and speaks well and is starting to write with the hand he could not, and he is with the speech therapist. The physiotherapy has been going very well. The only thing that is failing him is a little bit his memory…*

C3

*She is moving around a bit because she is eager to walk and go down to the street. She has a lot of willpower. She is always moving, but of course, she has the limitation she did not have before, right? I mean, for example, she can walk around the house with her walker. But we have to keep an eye on her…*

C1

### 3.5. Relevance for the Caregiver: They Are Not Alone

Some caregivers appreciate the involvement with this type of initiative with caregivers because they believe that they are people who are alone, that nobody trains them or gives them support to face a new situation with a lot of overload, and this changes their life completely. In addition, they believe that this also benefits the healthcare system.

*Those caregivers sometimes find themselves alone or helpless in circumstances (…) And of course, at that moment you say: How difficult can this person’s life be, right? I think we have to remember the caregivers, because that also benefits the healthcare system…*

C4

*Of course, support is needed for the caregivers because they do not prepare you for this…*

C2

### 3.6. Facilitating Factors for App Usage

Four subcategories were used to ascertain the causes and circumstances that have facilitated the use of the app by caregivers.

#### 3.6.1. Use of the App According to the Degree of Dependency

An interesting discourse reflected by one of the caregivers is the impact of the disease related to the use and/or need to use the app earlier or later in time. The statements reflect that people with a faster prognosis of recovery may find the app useful from the beginning, but people with a slower prognosis of recovery may find the app more useful over the months.

*A stroke does not affect everyone in the same way, because I understand that it is not the same if you have a stroke when you are 40 years old, if you are 82 years old, if you are 60 years old, or if you have previous pathologies or not. So, it is very difficult for you to know what will happen in each case. Although I consider that in my circumstances it may have been a little early because we are involved in a process that can take months and in the case of a young patient, who has the prospect of recovering much more quickly because he has also been caught earlier…, he will recover much sooner. Well, it is true that the moment at which you do it is correct. I understand that there must be hundreds of cases, right? I think it is better to err on the side of too early than too late…*

C4

#### 3.6.2. Digitally Competent Caregivers

All the caregivers interviewed used a cellphone, at least, for basic use, such as calling and messaging, in addition to consulting the internet, reading news, or shopping. This is indicative of the fact that most caregivers, with an average age, are digitally competent in the use of a cellphone. Therefore, a basic app for consulting information and messaging is not an impediment to its use.

*Yes, yes, yes, yes, the cellphone is like a computer, and I use it for everything…*

C3

*Yes, well, besides, because of my job I have to do part of my working day from my cellphone, so, well, I send emails, I use it like any other teenager, but I am able to do any kind of business on my cellphone…*

C4

*Let’s see, I use it to call, obviously… What I use it more for right now is chatting with my siblings. And then to look at the newspaper if I have a minute, I like to find out what’s going on in the world to browse the paper and that’s it. And few purchases, I can’t afford…*

C5

#### 3.6.3. App Design and Usability

Generally, caregivers who used the app found it easy and intuitive to use. Most of them emphasized that anyone with basic smartphone skills could use it without major issues.

*I thought it was easy. What I saw seemed easy, that’s for sure. I mean, to handle because I went in to look at some things when I was in the hospital, and it seemed like an easy application because there are some that are a bit of a mess, and it says whew, I’ll pass and the only thing I can tell you is that I found it a bit easy to handle…*

C1

*Yes, it was easy. I mean, if you have a little bit of intuition, it’s easy to use…*

C2

*No, it works well. If you have a little bit of basic telephone handling, which everybody has, I don’t see it as difficult…*

C4

#### 3.6.4. Expanded App Notifications

Users noted that they would like to be reminded of the event as soon as the app was offered to them, either in the form of notifications or by the managers. This is because in the first few months after the event, they have a lot on their minds and forget.

*If that is the case, I think it is very good that a few months have passed and that you contact me so that I know that, although I have not used it, is still there…*

C4

*For example, this happens to me with Sanitas, sometimes I get an email with information, healthy food and then you click on it, you know, because I’m going to click on it as a reminder, right? And you see a little bit if… maybe you remember that you have it or something and then you click on it out of curiosity, like a reminder that you have it…*

C1

*Maybe now that I’m talking to you I’ll bring the phone home so I can use it…*

C5

### 3.7. Source of Consultation in Case of Doubts and Reliability of Information

Most caregivers admitted having been informed through the app at some point. Also, most of them complemented it or were informed through the internet and/or the professionals who were close to them (in the hospital, where the patient was still recovering); therefore, they did not need to consult the information in the app too much.

The caregivers stated that they were aware that they could not trust all the information available on the internet and that they were grateful to have information collected by professionals. However, at the beginning, there were so many aspects to handle that it was easier for them to look on the internet or consult nursing and/or medical professionals whom they could trust. Therefore, they did not feel the need to look on their cellphone when they could ask a well-informed person.

*I learned quite a lot at the hospital because I spent practically a month there, and I learned quite a lot from what the nurses were doing because I applied myself quite a lot and then I didn’t need much more either…*

C2

*I appreciated it because, well, it is also a way of having the information biased in the same place where you can go and look for it, because at the beginning it was like well, I am going to look to see what is stroke, what are the consequences or… sometimes on the Internet you know that when you search you find what you do not want or what you want to see exactly things you want to see that maybe are not relevant or that is not about the disease itself…*

C5

*But it is true that when I have a doubt I have always turned to the people who are here, and I think it is silly to go to the Internet, don’t you? If you can raise your head and talk to a real person…*

C4

*I have looked at YouTube for more information, and I have been taking advice from there. Then, between what I’ve read on the Internet and the videos I’ve seen, I’ve been taking notes. I have also read articles on the Internet and then nursing videos. In addition, a nurse comes to visit me (…) and all the doubts I have had have also been solved…*

C2

### 3.8. Limitations on the Use of the App

To determine the limitations that caregivers encountered in gaining adherence with the app, this category was divided into three subcategories.

#### 3.8.1. Digital Burnout

On some occasions, there is a feeling of exhaustion with the digital environment. The use of digital devices to remotely cover work responsibilities, among others, with the overload of care and high levels of attention, makes the priority in the use of the phone restricted to what is strictly necessary.

This situation affects the use of a new app, even though it may be useful, as there is no adherence or time dedicated to it.

*But sometimes because of laziness, and also because I’m tired of my cell phone from working and then I don’t feel like going to the computer again and again…*

C1

#### 3.8.2. The Moment to Introduce the App Is Very Relevant

The moment of presenting or introducing the app to the caregivers seems a relevant and pressing topic. Most caregivers agreed that, although it was good to know that it was there, they did not need to consult it in the first months of the disease; this is because they either had many things to adapt to or they could find out what they needed from the professionals closest to them.

Therefore, they remarked that the app can be more useful when they are at home and have no one to turn to when they have questions.

*And I looked a little bit over, but I was still in the hospital. And I say, well, when I get home I’ll have more doubts and it will be more necessary…*

C1

*No, because I didn’t think it was necessary either. No, there wasn’t much to ask. Actually, since I had been informed by other places in the hospital, I didn’t see the need to ask about things that I had already been informed about. When I am at home, and suddenly I have a question and I no longer have the nurses, the doctors, the therapists next to me, then that will be the moment when I say look, I am going to remember the app, and then I am sure that it will solve things for me…*

C2

*You know what happens? In the first month or the first few days, there are many things to do looking for everything to go well and you don’t pay attention to the application, but I tell you that after that, yes, I did. When all that happened, I got home, I dropped the papers and honestly I didn’t pick it up again until… well it was my dad’s month, which was harder…*

C3

*I understand that it is an application more focused on the caregiver when it is in a more home context, right? And from a personal point of view, that is, in my specific case, yes, it may be a bit early… C4*

C4

#### 3.8.3. Prioritizing Rest and Focusing Attention on Technical and Compatibility Issues

The lack of time and the fatigue involved when assuming the role of caregiver are a recurring theme in the speeches of caregivers, who assume it as a handicap when using the app.

The learning curve for a new tool, while simple, involves time and concentration that, in their new role as a caregiver, does not allow them to adhere to it with assurance that it will become a much-needed application.

*When I go to my cellphone, I have the application there. What I don’t have time for, that’s it. And well, your entry at the beginning, but the truth is that I have almost no time for anything. So of course, sometimes I have to spend a little time wasting time on something, don’t you understand?*

C1

*It’s just that between the fact that I have a lot of busy time and sometimes I don’t have much time to think. I’m thinking all day long. I need this, I need that, I have to go shopping…*

C2

*These last two months I haven’t even remembered. Too many things on my mind, I guess, and I had totally erased…*

C5

*But since I’m not at home either. Now the last thing I want to do when I get home, when I’m exhausted, is to spend my time with stuff…*

C4

## 4. Discussion

The present study explores the perceptions and attitudes of informal caregivers of stroke patients in the context of *Stroke-CareApp*, a smartphone application created specifically for this population.

Following the conceptual principles of Heidegger’s phenomenology–hermeneutics, the understanding of the meaning of “care” for new caregivers is essential, since it is not possible to have an objective knowledge of the human being but an interpretation and description of the experiences, their cultural norms, and historical time [23,39].

First, the need for involvement of the rest of the family and/or external help to cope with the new situation after the stroke was observed. This coincides with other studies [40,41,42] that describe the initial difficulties and needs of informal caregivers, such as family and economic support, psychological support or training, and information on the process. Therefore, *Stroke-CareApp*, by providing training and information, and support to other caregivers, would be a complementary intervention to improve the situation of caregivers.

This involvement and cohesion of the family is important and, as described by Bourdieu, is ascribable to the existence of fusion forces, such as moral and affective ties, the exchange of gifts and services, or family celebrations. However, they may not occur or may be maintained over time because opposing forces of disintegration will appear due to individual interests or not wanting to submit to the family [29].

Noell-Boix et al. [43] identified how caregivers experience negative feelings and emotions when caring for their family members, as reflected by the participants in this study, who saw their social and family relationships diminished, along with a deterioration at work and economic levels. In addition, they verbalized being constantly tired and having the feeling of not having time for themselves. All of the above coincides with patterns characteristic of caregiver overload, as described by Ortiz et al. [44], who discussed the total physical, psychological, emotional, and financial cost of providing care to a person.

This overload is also related to the degree of dependence and the ease of recovery of the caregiver. The current study describes the difference between patients who are hospitalized at the time of the interviews and those who are not: caregivers who report feeling more liberated, satisfied, and with less involvement coincide with having their relative at home, which implies a lower degree of dependence. As evidenced by Ocampo [45] and Rodriguez [46], there is a direct relationship between the degree of dependence in basic activities of daily living and overload. Pinquart and Sörensen [47] highlight in their meta-analysis that physical and emotional overload, together with the high degree of dependence of the person being cared for, is consistently associated with poorer physical health in the caregiver.

In addition, Orem [30,48], in his theory of the “self-care deficit”, reflects on the relationship between the well-being of the caregiver and the state of health, number of tasks, and cognitive state of the caregiver.

In the new process of acquiring the role of caregiver, many negative aspects appear, as we see above. Nevertheless, there are also positive aspects, since caregivers describe that, despite all the effort involved in the new role, seeing that their effort and participation in caregiving reduce the recovery time of their family member comforts them. This aligns with the findings of other studies [49] describing the positive aspects of caregiving, such as satisfaction in helping others, greater self-confidence, and the development of empathy.

Another aspect to consider in the use of the app is the digital competence and ease of use of the application. This is because the population group of informal caregivers can include all ages, although different studies [50] place the majority above 45 years of age. In this study, the interviewees reported using a cellphone for tasks such as calling, messaging, reading news, or online shopping and affirmed that they had not encountered any problems when using the app and called it not too complex. This coincides with the INE study, whose findings demonstrated that people under 65 years of age, as well as the elderly, are accustomed to using these technologies and that the design and complexity of apps are the main barriers encountered by this population group [51].

Along these lines, several studies [52,53] show a growing trend toward the use of digital resources and mobile applications among caregivers. There are also authors [53,54] who emphasize that the use of these resources in older adults depends on factors such as self-efficacy, optimism, and available social networks, which reinforces the importance of the application in question being intuitive and adapted to different skill levels.

The access to collected information proposed by our app is a value taken into account by the participants, who say they know that they cannot trust everything they read on the internet. However, in spite of this, the caregivers interviewed have continued to consult other sources of information, such as the internet, in addition to the app. This aligns with studies that reflect a high percentage of health-related consultations on the internet by the population [51,55].

In addition, caregivers who have their family member admitted to the hospital in the first months after the episode agree that they have had little need to go to the app because they had professionals to ask and from whom to learn. This aspect follows the line of vicarious learning developed by Bandura [28], in which lessons are learned by observing what others do.

It is also necessary to focus on the limitations that caregivers have had in using the app. Although they believe that it was positive to know about the existence and benefits offered by the app, they did not make excessive use of it because, in these first months, they had many changes in their lives, making it difficult to accommodate other things. In addition, the doubts they had were resolved through other sources that were easier for them to consult. This observation coincides with those described in other studies [50], which describe how most of the changes in caregivers’ lives occur in the first year after the acquisition of the caregiving role.

This low usage in the first few months, as reported by participants due to the difficulty of integrating it into their routine and their preference for familiar sources, is supported by studies such as that by Mehra and Ling [52], which shows that caregivers value the immediacy of information and the ability to reduce social isolation, although they state that the lack of time, emotional overload, and doubts over the reliability of information are significant barriers to the use of technology.

These aspects, together with those highlighted in other studies [56,57], such as insufficient digital literacy, design, app interface, or concerns about privacy, have a negative influence on adherence to health apps.

Despite not having used the app as much as they would have liked, participating caregivers appreciated the researchers’ involvement with them, as they felt lonely and unsupported by others. As described by Melo [58], it is important to “care for the caregiver” and consider it a holistic process.

Finally, the systematic review by Boots et al. [59] confirms that the use of internet-based resources can improve caregivers’ knowledge and skills, but it also shows that the reduction in burden and stress is moderate and highly dependent on adherence. In addition, they highlight that participation tends to decrease if there are no motivational elements or human contact, which is in line with the findings of our participants, who recognize the benefits of the application but do not always incorporate it into their daily lives in a sustained manner.

Due to the phenomenological approach focused on exploring experiences in depth and the limited sample size, variables such as age, educational level, or socioeconomic background were not specifically analyzed. However, the literature shows that variables such as digital literacy, previous experience with technology, educational level, and cultural background significantly influence the adoption and continued use of mobile health applications [57]. Therefore, although our study did not include such analyses, and this could be considered a limitation, it would be advisable to incorporate them in future research to obtain a more complete and contextualized interpretation of the results.

On the other hand, for methodological reasons, data collection and some activities of the study itself depended on the use of the mobile application developed; therefore, it was decided to exclude caregivers with lower digital literacy, which leads to a significant limitation of the study in the inclusion criteria, thus introducing a selection bias that limits the transferability of the results to the entire caregiver population.

The possibility of recall bias must be considered, since, although the six-month delay between the presentation of the intervention and the interviews was intentional and part of the study design, so that participants could use the app continuously and cumulatively, it may also imply a certain risk of forgetting specific details.

Furthermore, due to the PI’s participation in different stages of the study, it is necessary to recognize the possibility that some responses may be influenced by social desirability, thus biasing the interpretation of the data, despite the reflective strategies implemented.

## 5. Conclusions

Exploring the perceptions, opinions, and attitudes of informal caregivers of stroke patients to the use of a new intervention based on a smartphone app created and designed specifically for them highlights the advantages and perceived potential of this intervention.

Because the conclusions drawn from this study are based on participants’ subjective perceptions rather than objective measurements, statements regarding the effectiveness of the intervention should be understood within that interpretive framework.

The digital competence observed in the participants in this study, together with an accessible design focused on their needs, makes *Stroke-CareApp* be perceived as an alternative for information, training, and direct contact with peers, which caregivers appreciate having at their fingertips. It is a way to feel less lonely and lost in a new high-stress situation with drastic changes in their lives.

However, it should be kept in mind that this intervention is a complementary aid and does not replace other external aids or the involvement of the rest of the family. Therefore, although its availability is suggested from the outset of the episode, some caregivers report feeling overwhelmed; accordingly, a gradual introduction, adapted to the level and disposition of each caregiver, should be taken into account. During this period of adaptation, it seems interesting to provide reminders and informative messages from the healthcare team.

In this sense, the app could represent a promising alternative in supporting informal caregivers of stroke patients, respecting their emotional context and pace of adaptation. Future research with larger samples and complementary methodologies addressing this phenomenon will allow for a more accurate assessment of the impact this app may have on the quality of life of these caregivers.

## Figures and Tables

**Table 1:** Sociodemographic data of participants.

Code	C1	C2	C3	C4	C5
Age (years)	55	55	56	25	49
Sex	Male	Female	Female	Female	Female
Educational level	Professional training	Baccalaureate	Professional training	Completed primary education	Higher education
Income level (EUR/month)	2000–3000	<500	<500	500–999	1000–1999
Cohabitation	Single	Spouse	Spouse and children	Father	Spouse and children
Residence	Urban	Urban	Urban	Urban	Urban
Type of housing	Rented	Ownership	Ownership	Ownership	Ownership
Employment status	Professional	Self-employed	Professional	Professional	Professional
Relationship with the patient	Father/Mother	Son/Daughter	Son/Daughter	Son/Daughter	Son/Daughter
Do you receive help with the care of your relative?	Yes	Other family members	Child	Yes	Yes
How much help (in hours per day) do you receive?	<1	>8	>8	>8	>8
Initial Barthel of the relative with stroke	20	20	25	10	15
Barthel at six months of the relative with stroke	25	30	40	40	30

## Data Availability

Due to the qualitative nature of the data and the fact that the full transcripts contain information that could identify the participants, they cannot be shared publicly. However, representative anonymous quotes supporting the findings are included in the manuscript. Researchers interested in accessing additional data (e.g., specific anonymized excerpts) may request them from the corresponding author. Access will be evaluated on a case-by-case basis, subject to ethical approval and the signing of a confidentiality agreement.
